# Hypertrophy of the Prostate in Dogs

**Published:** 1897-03

**Authors:** 


Hypertrophy of the Prostate. M. E. Lienaux has made certain observations on hypertrophy of the prostate, and gives the results of his observations in four cases, having made post-mortems on three of them, confirming his diagnosis:

Case I. *Hypertrophy of the prostate, with intermittent hemorrhages.*—A large Ulmer (Great Dane), about nine or ten years old, apparently strong and healthy: after micturition or exercise a slight amount of blood escapes from the penis. An examination of the prepuce, foramen, urethra, and the bladder does not reveal any diseased condition; but on making a digital examination per rectum the prostate was found much increased in volume, being about the size of an egg and very hard and tense; the blood passed was pure and contained a large number of spermatozoa. On passing the catheter the urine is found to be clear and not subsequently followed by the passage of blood; hemorrhage being caused by the enlargement of the prostate, blood is only passed when there has to be some pressure exercised to pass the urine. Palliative treatment was advised and greatly relieved the symptoms, but six months afterward there was complete retention of the urine; it being impossible to pass the catheter, the author took away the urine with a trocar (cystopaxie). The animal died a short time after the operation. On post-mortem the ordinary changes present in uræmia were found, and also considerable enlargement of the prostate. The gland was the shape of two nuts, the two lobes being entirely separated; the structure was homogeneous and soft, being a case of simple hypertrophy.

CASE II. *Cystic calculi following hypertrophy of the prostate.*—The dog, seven years old, had hæmaturia for several months, followed by retention of urine. On examination a calculus was found in the groove of the os penis; on performing urethrotomy the stone was removed and the bladder emptied; while doing so about a dozen small stones were passed and a great amount of sediment. The owner having abandoned the animal, it was destroyed and an autopsy was held. Sixteen small calculi about the size of small peas, spherical in shape and smooth and glossy, were found in the bladder. Chemically they were composed of phosphate of ammonium and magnesium. The prostate was greatly enlarged, being 150 mm. in circumference.

CASE III. *Perineal hernia and hypertrophy of the prostate.*—A very large dog, aged twelve years, had an enlargement beneath the anus on the right side, which varied in size; if pressed suddenly it was easily reduced, but after continued manipulation it became very hard; the animal passed urine with difficulty. A diagnosis of hernia of the bladder was made; an opening was made through the perineum upon the bladder, and by that means it was reduced. Some time afterward the same condition recurred and was treated in a similar manner, but when it occurred the third time it was necessary to perform cystotomy. The animal died a short time afterward from hemorrhage, consequent on the operation. On post-mortem a very large prostate was found.

CASE IV. *Hypertrophy of the prostate, with hydronephrosis.*—This was a case of hydronephrosis, and on post-mortem there was found to be broncho-pneumonia and also decided hypertrophy of the prostate. This last anomaly was the only obstruction that interfered with urination. The prostate measured 150 mm. in circumference.

*Summary.* It is very easy to understand the causes of these diverse alterations. The prostate, being hypertrophied, presses on the rectum and on the urethra, causing an interference to the free passage of urine and the feces. The interference to the passage of urine causes a hypertrophy of the muscular coat of the bladder to compensate for the obstruction; this is sufficient at first, but as the disease goes on and the pressure becomes greater constant retention is followed by paralysis of the muscular coat of the bladder, inflammation, or sclerosis, and finally the formation of a calculus. The hemorrhage is produced as a consequence of the hypertrophy of the gland. The pressure constantly exercised on the vesico-prostatic circulation interferes with the circulation, causing congestion and hyperæmia, and hemorrhage is caused by the great effort made in urination. The hernia of the bladder is caused by the bladder being greatly distended and causing relaxation of its attachments, making it extremely mobile, and when the animal makes constant efforts to urinate it is forced out of position by the great pressure. The treatment of hypertrophy is either palliative, by means of laxatives, fomentations, etc., or castration as a radical treatment.—*Annales de Médecine Vétérin.*, 1896.

